# Fear of weight gain during cognitive behavioral therapy for binge-spectrum eating disorders

**DOI:** 10.1007/s40519-023-01541-8

**Published:** 2023-03-06

**Authors:** Rachel M. Butler, Elizabeth Lampe, Claire Trainor, Stephanie M. Manasse

**Affiliations:** 1 Department of Psychological and Brain Sciences, University of Louisville, Louisville, KY USA; 2 Department of Psychology, Drexel University, Philadelphia, PA USA; 3 Center for Weight, Eating, and Lifestyle Science, Drexel University, Philadelphia, PA USA

**Keywords:** Eating disorders, Binge eating disorder, Bulimia nervosa, Fear of weight gain, Cognitive behavioral therapy

## Abstract

**Purpose:**

Fear of weight gain may play a central role in maintaining eating disorders (EDs), but research on the role of fear of weight gain during cognitive behavioral therapy (CBT-E) for binge-spectrum EDs is sparse. We examined changes in fear of weight gain during CBT-E for binge-spectrum EDs. We investigated whether fear of weight gain predicted loss of control (LOC) eating or weight change.

**Methods:**

Participants (*N* = 63) were adults of any gender recruited as part of a larger trial. Participants received 12 sessions of CBT-E, completed diagnostic assessments at pre-, mid-, and post-treatment, and completed brief surveys before sessions.

**Results:**

Fear of weight gain decreased across treatment, moderated by diagnosis. Those with bulimia nervosa spectrum EDs (BN-spectrum), compared to binge eating disorder, reported higher fear of weight gain at baseline and experienced a larger decrease in fear across treatment. Those reporting higher fear of weight gain at a given session experienced more frequent LOC episodes the following week. Fear of weight gain was not associated with session-by-session changes in BMI.

**Conclusion:**

CBT-E results in decreases in fear of weight gain, but levels remain high at post-treatment, especially for those with BN-spectrum EDs. Future interventions should consider targeting fear of weight gain as a maintaining factor for LOC episodes

**Trial registration:**

NCT04076553.

**Level of evidence:**

Level II controlled trial without randomization.

## Introduction

Eating disorders (EDs) are severe psychiatric illnesses characterized by disrupted consumption of food (e.g., restrictive or loss of control [LOC] eating) and often involve overvaluation of one’s shape and weight, a drive for thinness, and body image disturbances [1]. Specifically, bulimia nervosa (BN) and binge eating disorder (BED) involve binge eating episodes in which an individual consumes an objectively large amount of food in a relatively short amount of time and experiences a sense of loss of control [1, 2]. Those with BN also engage in compensatory behaviors (e.g., purging, laxative use, excessive exercise) to counteract the effects of a binge episode [1]. Cognitive behavioral therapy for EDs (CBT, including an enhanced version, CBT-E) is a front-line treatment for BN and BED; however, only about 30–50% of individuals achieve remission of ED symptoms following treatment [3, 4]. Considering that nearly half of patients have suboptimal treatment outcomes from the gold-standard treatment, further investigation of maintenance factors in EDs, and the effects of CBT-E on these specific factors is warranted.

A clearer understanding of maintaining factors in binge-spectrum EDs would allow for development of more targeted, specific interventions. Fear-based maintaining factors in EDs, such as fear of weight gain or “fatness” and its consequences,^1^ are potential intervention points. Fear of weight gain is a central symptom for many individuals with EDs [5] and has been found to predict worse ED pathology in a sample with anorexia nervosa [6]. In fact, fear of weight gain is theorized to be a core ED symptom transdiagnostically, including for those with BED [7, 8]. Additionally, in a sample seeking weight loss, those with BED had significantly higher fear of weight gain than those without BED, regardless of weight status [9].

Fear of weight gain may originate from an overvaluation of one’s shape and weight—a core symptom across binge-spectrum EDs [5, 8]. As a person prioritizes having an “ideal” shape or weight over other aspects of their identity, fear of weight gain may motivate disordered eating behaviors [10]. Further, learning theory suggests that fears may arise in EDs as unwanted outcomes (e.g., social rejection) are paired with a stimulus (e.g., weight gain), thus reinforcing the learned relationship between weight gain and fear [11, 12]. Those with EDs fear negative outcomes of weight gain, including social consequences (e.g., being rejected), personal consequences (e.g., being “lazy”, losing control over life), physical sensations, and social eating [13, 14].

In addition to being a central symptom for EDs, fear of weight gain may also be motivating disordered eating behaviors through a cycle of avoidance, as is seen in anxiety disorders [10]. Individuals with a fear of weight gain tend to avoid foods they believe will lead to weight gain [12, 15], develop ritualized eating behaviors, and engage in body checking or avoidance in an attempt to minimize risk of weight gain. As in other anxiety-based disorders, these avoidance behaviors actually strengthen fears and perpetuate the fear and avoidance cycle [16]. For example, restrained eating patterns and food and body avoidance contribute to binge eating episodes in binge-spectrum EDs. Additionally, for those with BN, compensatory behaviors (e.g., purging, excessive exercise, laxatives) serve as an effort to reduce fear of weight gain following binge episodes and maintain the binge/purge cycle [2, 10]. As individuals experience fear about changes in their weight, they are driven to engage in behaviors to avoid weight gain and associate these avoidance techniques with weight stability—they fear that if they were to abandon those behaviors, weight gain would occur and, with it, feared social and personal consequences [10, 17]. Although many of these behaviors occur to varying degrees for those with BED (e.g., body avoidance, dysregulated and restrained eating patterns), further exploration of the relevance of fear of weight gain in maintaining binge eating for those with BED is needed.

Theory supports conceptualization of EDs using an anxiety-based model, but little research exists on the temporal relationship between fear and disordered eating behaviors. For example, fear of weight gain may lead to restrictive eating, thus resulting in LOC episodes. Similarly, fear of weight gain may increase attention to threat (e.g., foods high in fat content), leading to an attempt to avoid these foods followed by a binge episode containing these foods. A better understanding of this link would clarify whether targeting fear of weight gain directly in treatment should result in decreases in disordered eating behaviors. Additionally, many patients with EDs place value on fear of weight gain, as they believe it will lead to achievement of the weight loss goal and/or protect them from the feared outcome of weight gain [18]. Individuals may believe that a reduction in fear would result in subsequent weight gain, and they are disinclined to challenge its validity. A better understanding of the relationship between fear of weight gain and actual weight changes over the course of treatment may help clinicians garner buy-in for confronting fear of weight gain in treatment.

CBT-E does not specifically or explicitly target fear of weight gain. Although CBT-E is not an exposure-based treatment—such as the first-line interventions for targeting fears in other populations (e.g., anxiety, OCD)—there are a number of components to CBT-E that may inherently require confrontation of fears [19]. CBT-E involves regularizing eating patterns, which may force some to confront fears that eating differently will result in weight gain. A complement to regular eating is weekly open weighing, which allows the individual to gain disconfirming evidence that, in fact, feared weight gain as a result of changing eating patterns does not occur [20]. Further along in CBT-E, individuals engage in dietary rule breaking experiments, which allow them to break rules surrounding eating and observe outcomes. These processes violate expectancies about feared outcomes and likely assist in decreasing fear of weight gain. In a study of individuals with anorexia nervosa, decreases in fear of weight gain occurred during CBT and were predictive of improvements in dietary restraint [21]. Research has yet to examine whether CBT-E results in changes in fear of weight gain for adults with BN or BED. Additionally, given that those with BN engage in significantly more behaviors aimed at avoiding feared weight gain (e.g., purging, excessive exercise, laxative use), it will be important to understand whether those with BN experience differential changes in fear of weight gain compared to those with BED across treatment.

The current study sought to clarify whether CBT-E produces changes in fear of weight gain and to better understand whether fear of weight gain is predictive of disordered eating behaviors and treatment outcomes during 12 sessions of modified CBT-E for binge-spectrum EDs. Modified CBT-E included self-monitoring, regular eating, overvaluation of weight/shape, reducing restriction/restraint, and addressing mood-related changes to eating. We predicted that (1) fear of weight gain would decrease over the course of treatment given the inherent confrontation of fears involved in CBT-E, (2) changes in fear of weight gain over treatment would be moderated by diagnosis (i.e., BN-spectrum, BED), (3) within-person increases in fear of weight gain would be associated with higher frequencies of LOC eating episodes in the following week, and (4) between-person, those with a higher fear of weight gain would experience a higher frequency of LOC episodes. Additionally, many individuals with EDs hold the metacognitive belief that their fear of weight gain is critical to evading actual weight gain. To understand whether fear of weight gain was actually associated with weight change, we explored the bidirectional relationship between fear of weight gain and BMI within- and between-person across treatment.

## Methods

### Participants

We recruited adults with clinically significant binge-spectrum EDs (*N* = 63), including BN-spectrum and BED, from the community for participation in a parent trial of CBT-E augmented by inhibitory control training (clinicaltrials.gov identifier: NCT04076553). Participants were included in the parent trial if they were between 18 and 55 years old, experienced an average of at least one objective binge eating episode per week over the previous 12 weeks, had stable psychiatric medication for the past 3 months (if applicable), had a reliable Internet connection, and were located in the USA and willing and able to participate in remote intervention and assessments. Participants were excluded if they were not fluent in English, were below a BMI of 18.5, were planning to begin (in the next 6 months) or currently participating in another weight loss treatment or psychotherapy for binge eating and/or weight loss. Participants were not eligible if they had a diagnosis of autism spectrum disorder or intellectual disability, were currently experiencing other severe psychopathology that would limit their ability to engage in the treatment program (e.g., severe depression, substance dependence, active psychotic disorder), or demonstrated high levels of inhibitory control (and thus would not benefit from the inhibitory control training portion of the treatment). Participants were also excluded from the parent trial if at least half of their binge episodes were composed nearly entirely of fruit/vegetables (i.e., 80% or more of the total food consumed during binges were raw fruits and vegetables) because of the intention to test inhibitory control training toward more traditional binge foods (e.g., pizza, ice cream). Participants were not eligible if they had experienced a recent head trauma, neurological condition, or brain condition that would interfere with completion of daily computer trainings.

The current study represents a secondary analysis of data from the parent trial. For the current study’s analyses, participants were included if they completed at least one session of treatment. Of these 63 individuals, 11 (17.5% of sample) dropped out of treatment prior to completing all 12 sessions. Attrition rates in our study are comparable to rates in other trials of CBT for EDs [3]. Participants completed 10.48 treatment sessions on average (SD = 3.51). In the current sample, 29 participants were randomized to the “sham” training condition, and 34 to the inhibitory control training condition. Table 1 depicts participant demographic information, diagnoses, and BMI.

**Table 1:** Participant demographics and baseline characteristics by diagnosis (*N* = 63)

	Bulimia nervosa (incl. subthreshold; *n* = 27)		Binge eating disorder (*n* = 36)	
	Mean or *n*	SD or %	Mean or n	SD or %
Age	37.2	12.7	42.9	9.6
Gender				
Male	1	3.7%	6	16.7
Female	26	96.3%	30	83.3
Race				
White	21	77.8%	31	86.1%
African American	2	7.4%	4	11.1%
Asian	2	7.4%	0	0%
Multiracial	1	3.7%	1	2.8%
Unknown/prefer not to say	1	3.7%	0	0%
Ethnicity				
Hispanic/Latinx	4	14.8%	3	8.3%
Non-Hispanic	23	85.2%	33	91.7%
Disordered eating behaviors				
Binge episodes past 3 months	91.4	58.9	91.3	48.6
Compensatory behaviors 3 months	71.7	77.3	0.5	1.4
BMI	31.3	8.3	36.2	12.6
Fear of weight gain				
GFFS 10-item scores	30.9	5.7	23.8	7.4

BMI body mass index, *GFFS* Goldfarb Fear of Fat Scale

### Procedures

#### Recruitment and assessments

Participants were recruited to participate in a larger randomized controlled trial (RCT) of inhibitory control training adjunct to CBT-E for binge-spectrum EDs. The current sample includes all participants recruited for the RCT. Recruitment methods included radio and social media advertising. Interested individuals completed a phone screen to assess initial eligibility before being invited for a baseline assessment to determine final eligibility. Assessments were conducted by independent trained evaluators at pre-treatment, after session 4 (“mid-treatment”), after session 12 (“post-treatment”), and at 3-month follow-up. The current study utilizes data from pre-treatment, mid-treatment, and post-treatment assessments. Participants also completed short online surveys before each CBT-E session. Participants received free treatment and were compensated $100 for completing all assessments and up to $100 for inhibitory control trainings. Procedures were approved by the Institutional Review Board and informed consent was obtained from all participants.

#### Treatment

All participants received modified 12-session CBT-E. We modified the original CBT-E manual (focused version; the default version focused exclusively on eating disorder psychopathology) based on Fairburn [19] to be delivered in 12 weekly individual sessions. Participants completed a 120-min intake (Session 1), and all following sessions were 60 min. CBT-E consisted of self-monitoring, regular eating (focused on reduction of dietary restraint), urge management strategies, discussion of overvaluation of weight and shape, dietary rule breaking experiments, and addressing event and mood-related triggers to binge eating. Elements of the complex broad version of CBT-E such as reducing perfectionism and improving low self-esteem were omitted due to the time constraints of only 12 sessions. Otherwise, there was close correspondence between Fairburn’s focused CBT-E and the modified version delivered in this trial, it was simply compressed into fewer sessions. Participants were given homework (e.g., self-monitoring, regular eating goals, reducing shape checking, etc.) between sessions. Each session consisted of a review of homework, in-session weighing, content addressing one of the above treatment targets (e.g., overvaluation of weight/shape), and assignment of homework. The treatment was delivered by graduate student clinicians who were supervised weekly by licensed clinical psychologists. Due to the COVID-19 pandemic, CBT-E sessions were conducted in-person weekly prior to March 2020 and remotely via videoconference after March 2020.

Participants were also randomized to complete a 10-min computerized inhibitory control training or a sham training daily for the first four weeks, then once weekly for the duration of the treatment. These trainings were an adjunct to CBT-E and did not impact the CBT-E intervention. The inhibitory control training aimed to increase inhibitory control toward food items via a Go/No Go Task in which participants were presented visual food or non-food stimuli and instructed to respond as quickly as possible except when a “no go signal” (e.g., a blue circle) appeared, which was always paired with stimuli representing the participant’s self-reported binge foods [22]. The sham condition contained the same stimuli and instructions, but there were no “no go” signals (i.e., participants responded as quickly as possible to every stimuli), which was meant to serve as an attention control for the inhibitory control training. Participants completed these trainings online at home on their personal computers.

### Measures

#### Eating pathology

The Eating Disorders Examination 17.0 (EDE) [23] was used to assess disordered eating symptoms over the previous 3 months. The EDE is a well-validated, semi-structured diagnostic interview. Based on EDE interviews, individuals were assigned a diagnosis of BN, “low-frequency BN” (i.e., other specified feeding and eating disorder), or BED using behavioral criteria based on DSM-5 frequencies for diagnoses: BN (*n* = 22) was defined by 12 or more objective binge episodes and 12 or more compensatory behaviors in the past 3 months, low-frequency BN (*n* = 5) was defined by 12 or more objective binge episodes and between 6 and 11 compensatory behaviors in the past 3 months, and BED (*n* = 36) was defined as having had at least 12 objective binge episodes and fewer than 6 compensatory behaviors in the past 3 months. Research has demonstrated that there is limited clinical utility in the distinction between sub- and full-threshold BN Johnson et al., [24]; thus, for our analyses, we merged individuals with BN and “low-frequency BN” into one group: BN-spectrum.

#### Fear of weight gain

The Goldfarb Fear of Fat Scale (GFFS; [25] is a 10-item measure that was used to assess fear of weight gain or “fatness” at pre-treatment, mid-treatment, and post-treatment assessments. Items are rated on a 4-point scale from 1 (“very untrue”) to 4 (“very true”), with scores ranging from 10 to 40 on the full scale. To reduce participant burden, we selected four items from the GFFS to administer prior to each therapy session, with possible scores ranging from 4 to 16. These items were: “*My biggest fear is becoming fat*,” “I am afraid to gain even a little weight,” “Becoming fat would be the worst thing that could happen to me” and “If I eat even a little, I may lose control and not stop eating”. The GFFS is a reliable and well-validated measure of fear of weight gain among individuals with EDs and has been shown to differentiate weight-related fears in clinical and non-clinical samples [25]. Internal consistency of the ten-item measure in the current sample at pre-treatment was good (*α* = 0.88). The four-item measure demonstrated acceptable internal consistency at session 1 (*α* = 0.75).

#### Loss of control episodes

Prior to each session, participants were asked to report frequency of binge eating or LOC episodes over the past 7 days. Participants were asked “How many times have you felt a sense of loss of control over your eating?” The items did not distinguish between objective and subjective binge episodes, and thus the weekly measure of LOC episodes likely included a range of sizes of LOC episodes.

#### Body mass index (BMI)

Session-level BMI was calculated using participants’ self-reported height (given at baseline) and a weight obtained (in pounds). Weight was collected by the therapist during in-person sessions and was reported by the participant during sessions that occurred via videoconference. Baseline BMI was obtained using participant-reported height and a weight obtained (in pounds) by the assessor at the pre-treatment assessment.

### Data analytic plan

Data were analyzed using SPSS Version 26 (IBM [26] and R [27]. We ran linear models using multilevel modeling (MLM) due to the nested nature of our longitudinal data (observations within person). In all analyses, we included fixed predictor variables and the random intercept of person. We used restricted maximum likelihood estimation to handle missing data, which constituted 8.78% of session data and 7.3% of assessment data.

Time-varying predictors were separated into between-person effects which were grand mean centered (mean aggregate across the sessions) and within-person effects which were person-mean centered (deviation from participant’s mean at each session; raw—mean). Time (session number or assessment point) was included in each model as a predictor.

We examined whether fear of weight gain changed throughout treatment using session-by-session data. We conducted a MLM with session number (i.e., 1–12) as a fixed effect predicting four-item GFFS scores. Additionally, we examined whether fear of weight gain using the full scale (10-item GFFS) changed across the pre-treatment, mid-treatment, and post-treatment assessment points. We conducted an MLM with assessment time point (i.e., 1, 2, 3) as the fixed effect predicting GFFS scores.

We examined whether the change in fear of weight gain (4-item GFFS) session by session was moderated by diagnosis of BN-spectrum compared to BED. We conducted an MLM with session (i.e., 1–12), diagnosis (BN-spectrum, BED), and the interaction between session and diagnosis as fixed effects predicting session GFFS scores. We also examined whether the change in fear of weight gain (10-item GFFS) during treatment was moderated by diagnosis. We conducted an MLM with assessment point (i.e., 1, 2, 3), diagnosis (BN-spectrum, BED), and the interaction between assessment point and diagnosis as fixed effects predicting GFFS scores.

We conducted an MLM to examine whether GFFS scores were predictive of LOC episode frequency. We used a lagging procedure such that GFFS scores at a session “t” predicted LOC episode frequency (patient-reported) at session “t + 1”. This lagging procedure allows for testing the direction of effects, in other words, fear of weight gain at a given week predicting the following week’s LOC episode frequency. This MLM included the fixed effect of GFFS scores at session “t” predicting LOC episodes at session “t + 1”.

We conducted MLMs to investigate whether BMI predicted fear of weight gain. We lagged GFFS scores in order to test whether BMI at session “t” (fixed effect) predicted GFFS scores at session “t + 1”. Conversely, we examined whether GFFS scores at session “t” (fixed effect) predicted BMI at session “t + 1”.

As a sensitivity analysis, we included computerized training condition (i.e., sham, inhibitory control training) in all models as a control, but findings remained consistent. Thus, we reported results of analyses without controlling for training condition. Cohen’s *d* effect sizes were calculated by transforming t-statistics.

## Results

### Baseline associations

We examined whether demographic characteristics (age, gender, ethnicity, race, and BMI) were associated with GFFS scores at baseline. Age was not correlated with GFFS scores, *r* = − 0.09, *p* = 0.50. Female participants had significantly higher GFFS scores at baseline, *t* (60) = 2.09, *p* = 0.04. Those who identified as Latinx or Hispanic did not have significantly different GFFS scores than those who did not, *t* (60) = 0.36, *p* = 0.72. GFFS scores were not significantly different between racial identities, *F* (4, 57) = 0.91, *p* = 0.46. BMI and GFFS scores were not correlated at baseline, *r* = − 0.06, *p* = 0.67. Those with BN-spectrum disorders had higher GFFS scores at baseline than those with BED, *t* (60) = 4.08, *p* < 0.001, *d* = 1.05. Past month binge episode frequency and BMI were not significantly different between those with BN-spectrum and BED, *p*s > 0.09.

### Change in fear of weight gain during treatment

GFFS scores decreased significantly over the course of treatment from session 1 (*M* = 11.32 *SD* = 2.97) to session 12 (*M* = 8.64, *SD* = 3.54), *B* = − 0.24, *SE_B* = 0.02, *β* = − 0.07, *t* (591.92) = − 14.05, *p* < 0.001, *d* = 1.16.

GFFS scores decreased significantly across treatment at assessment points, *B* = − 2.47, *SE_B* = 0.37, *β* = − 0.30, *t* (106.56) = − 6.61, *p* < 0.001, *d* = 0.93. Pairwise comparisons revealed that GFFS scores did not decrease pre-treatment (*M* = 26.89, SD = 7.53) to mid-treatment (*M* = 25.52, SD = 8.14), *B* = − 1.31, *SE_B* = 0.71, *β* = − 0.16, *t* (104.89) = − 1.84, *p* = 0.07, *d* = 0.26, but decreased significantly from pre-treatment to post-treatment (*M* = 21.90, SD = 8.31), *B* = − 4.99, *SE_B* = 0.74, *β* = − 0.61, *t* (105.33) = − 6.76, *p* < 0.001, *d* = 0.94. See Table 2 for descriptive statistics for outcome variables across treatment.

**Table 2:** Descriptive statistics of study variables across treatment

	BMI	LOC episodes past week	GFFS 10-item	GFFS 4-item
	Mean (SD)	Mean (SD)	Mean (SD)	Mean (SD)
Assessments				
Baseline	34.14 (11.18)	–	26.89 (7.53)	10.82 (3.30)
Mid-treatment	34.36 (11.69)	–	25.52 (8.14)	10.09 (3.37)
Post-treatment	34.00 (11.78)	–	21.90 (8.31)	8.72 (3.41)
Sessions				
1	–	5.07 (4.69)	–	11.32 (2.97)
2	–	4.65 (3.85)	–	11.44 (3.34)
3	35.71 (11.95)	2.89 (2.62)	–	10.80 (3.56)
4	34.61 (11.64)	2.42 (2.20)	–	10.37 (3.29)
5	34.13 (11.85)	1.74 (2.23)	–	10.06 (3.47)
6	34.32 (11.78)	1.85 (2.78)	–	10.16 (3.22)
7	34.77 (11.98)	1.35 (2.04)	–	9.87 (3.44)
8	34.44 (11.90)	1.35 (2.02)	–	9.78 (3.54)
9	34.87 (12.01)	1.10 (1.39)	–	8.94 (3.30)
10	34.14 (11.42)	1.17 (1.96)	–	9.44 (3.35)
11	33.73 (11.99)	0.43 (0.76)	–	8.86 (3.56)
12	35.73 (12.63)	0.67 (1.02)	–	8.64 (3.54)

There is no BMI from sessions 1 and 2 because weight was first collected in CBT-E session 3

*BMI* body mass index, *GFFS* Goldfarb Fear of Fat Scale

### Change in fear of weight gain moderated by diagnosis

Those with BN-spectrum tended to have higher GFFS scores, *B* = 2.17, *SE_B* = 0.77, β = 0.63, *t* (69.99) = 2.81, *p* < 0.01, *d* = 0.39. Diagnosis did not moderate the change in GFFS scores over time, *B* = 0.03, *SE_B* = 0.03, β = 0.01, *t* (591.29) = 1.02, *p* = 0.31, *d* = 0.14.

**Fig. 1:**
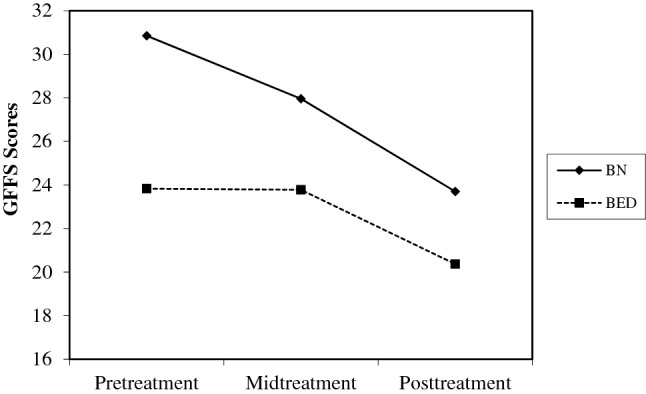
Change in fear of weight gain at assessments moderated by diagnosis. GFFS = Goldfarb Fear of Fat Scale, 10-item. Possible scores range from 10 to 40

Again, those with BN-spectrum tended to have higher GFFS scores, *B* = 8.71, *SE_B* = 2.26, *β* = − 1.06, *t* (124.14) = 3.85, *p* < 0.001, *d* = 0.54. Diagnosis moderated the change in GFFS scores over time such that those with BN-spectrum had greater decreases in GFFS scores over the course of treatment than those with BED, *B* = 2.07, *SE_B* = 0.72, β = − 0.25, *t* (105.50) = 2.85, *p* < 0.01, *d* = 0.40 (see Fig. 1).

### Fear of weight gain predicting LOC eating

We found between-person effects of GFFS scores on the patient-reported LOC frequency, *B* = 0.27, *SE_B* = 0.07, *β* = 0.30, *t* (51.40) = 3.99, *p* < 0.001, *d* = 0.54, such that those who had higher fears of weight gain (compared to other participants) at a given session reported more LOC episodes at the next session. We found no within-person effect of GFFS scores on patient-reported LOC episode frequency, *B* = − 0.01, *SE_B* = 0.08, *β* = − 0.01, *t* (581.34) = − 0.07, *p* = 0.96, *d* = 0.02, indicating that higher fear of weight gain (compared to one’s own norms) was not predictive of LOC episode frequency.

### Session BMI and fear of weight gain

We found no within-person effects of BMI on GFFS scores, *B* = 0.05, *SE_B* = 0.07, *β* = 0.17, *t* (402.30) = 0.71, *p* = 0.48, *d* = 0.10, and no between-person effects, *B* = 0.03, *SE_B* = 0.03, β = 0.09, *t* (54.03) = 0.80, *p* = 0.43, *d* = 0.14. These findings suggest that having a higher BMI (compared to the group mean or compared to one’s own mean) at a given session did not predict fear of weight gain at the following session.

We found no within-person effects of GFFS scores on BMI, *B* = 0.02, *SE_B* = 0.05, β = 0.01, *t* (400.06) = 0.42, *p* = 0.67, *d* = 0.06. There were also no between-person effects of GFFS scores on BMI, *B* = 0.53, *SE_B* = 0.51, *β* = 0.16, *t* (52.95) = 1.04, *p* = 0.30, *d* = 0.15. These findings suggest that having higher GFFS scores (compared to the group mean or compared to one’s own mean) did not predict BMI at the following session.

## Discussion

The current study was the first to our knowledge to examine fear of weight gain during CBT-E for the treatment of binge-spectrum EDs. Notably, we found that fear of weight gain decreased over the course of CBT-E, both in session-by-session ratings and from pre- to post-treatment assessment. These findings are encouraging, as CBT-E does not explicitly target this fear even though it is posited to be a maintaining factor of disordered eating [10]. We also found that those with BN-spectrum EDs began treatment with higher fear of weight gain and experienced greater reduction in fear across CBT-E. Our findings suggest that those with higher fear of weight gain at a given session experience more frequent LOC episodes in the following week, indicating a temporal link between this fear and LOC episodes. Finally, we noted that BMI and fear of weight gain were not associated during treatment, suggesting that changes in an individual’s weight do not correlate with fear of weight gain.

Despite decreases in fear of weight gain, fear remained somewhat elevated at the end of treatment (in particular, for those with BN-spectrum EDs) compared to scores reported in prior research with non-ED samples [28]. Exploration of which components of CBT-E contribute to decreases in fear of weight gain would allow for clinicians to emphasize certain components of treatment for individuals with particularly salient fears. Additionally, continued research into exposure-based treatments—the first-line intervention for addressing fears in other populations (e.g., anxiety, OCD)—may be a worthwhile approach for targeting fear of weight gain. These approaches include targeting food avoidance using exposures to feared foods and imaginal exposure to address the feared social and personal consequences (i.e., loss of identity, disgust, discomfort in one’s body) as a result of weight gain [29–31]. These exposure-based interventions have demonstrated marked improvements in disordered eating-related fears including fears of weight gain [30, 31]. Further investigation into exposure-based approaches to the treatment of binge-spectrum disorders is warranted if we hope to produce tangible changes in the fear of weight gain through treatment.

Both those with BN-spectrum EDs and BED started treatment with elevated levels of fear of weight gain compared to non-ED samples in prior research [28, 32]. GFFS scores for those with BN in the current sample were similar to previously published BN samples (*M* = 33.2; [28]. Individuals with BN (including low frequency) had higher fear of weight gain and experienced greater decreases in that fear over the course of treatment than those with BED. This suggests that those regularly engaging in compensatory behaviors experience higher fears of weight gain, possibly leading individuals to seek out compensatory behaviors as a method of mitigating the fear. Additionally, compensatory behaviors reinforce the fear, because individuals are prevented from learning that feared outcomes do not occur, and lack of weight change is associated with the use of the compensatory behavior [10]. CBT-E specifically assists patients in stopping compensatory behaviors [19], which may account for the finding that those with BN-spectrum EDs experienced a greater decrease in fear over the course of treatment as they relinquished use of these behaviors. Unfortunately, we were unable to effectively examine the relationship between fear of weight gain and compensatory behaviors due to the diagnostically mixed nature of our sample (i.e., high variability and a positively skewed distribution of compensatory behaviors). Future research should investigate the causal link between fear of weight gain and compensatory behaviors for those with BN.

Those with a higher fear of weight gain experienced more LOC episodes in the following week. Fear of weight gain appears to be a maintaining factor for LOC eating, even during treatment, such that individuals who are experiencing a higher level of fear are more at risk for a LOC episode shortly thereafter. This may occur through a pathway of restrictive or restrained eating. On the other hand, experiencing less intense fear of weight gain may be somewhat protective in that it puts individuals at lower risk for LOC episodes. This corroborates the theory suggesting that fears of weight gain are a core maintaining factor in EDs, and that targeting the fear may reduce symptoms [5, 10]. Alternatively, those with higher fear of weight gain may perceive more eating episodes as loss of control episodes than those who experience less intense fear of weight gain. We did not find within-person effects, which may be a result of lower within-person variability in fears of weight gain session-by-session. Altogether, our preliminary findings suggest that fear of weight gain may play a causal role in LOC eating, and future research should continue to explore this as a maintaining factor.

Interestingly, we found that BMI was not associated with fear of weight gain at pre-treatment, which adds to previous findings from a sample with anorexia nervosa [21]. Further, BMI was not predictive of fear of weight gain, nor was fear of weight gain predictive of BMI. This finding is highly clinically relevant, as many individuals with EDs believe fear of weight gain to be protective of actual weight gain, such that they are often ambivalent or reluctant to challenge the fear. The fact that BMI does not change as fear of weight gain decreases suggest that the fear is, in fact, not protective. Clinicians may consider using this information as a form of psychoeducation for patients when discussing the importance of confronting their fear. It is important to consider that our sample included individuals with binge-spectrum EDs who were not at a significantly low weight, so weight gain was typically not an explicit treatment goal. Future research should examine the association between BMI and fear of weight gain in a sample of low weight individuals who are undergoing weight restoration during CBT-E.

### Strengths and limits

Examining fear of weight gain at each session allowed us to predict the following week LOC episodes to establish a temporal association between fear of weight gain and LOC. Limitations include a somewhat narrow sample, as participants were ineligible to participate if they had high inhibitory control due to the intention to test inhibitory control training in the broader RCT. Additionally, although it would have been optimal to calculate clinically significant change in fear of weight gain, we were unable to do so given lack of norms for the GFFS and high variability in GFFS scores at pre-treatment (SD = 7.5, ranging 10–40). It will be critical to examine whether the changes in fear of weight gain during CBT-E are clinically significant, or whether additional methods of targeting fear of weight gain during treatment (i.e., exposure therapy; [17]) must be incorporated for those with more intense fears to attain significant improvements. Four items from the GFFS were administered at pre-session surveys, which were selected from a well-validated measure and intended to reduce participant burden. Additionally, prior research on fear of weight gain has tended to measure the construct using single items pulled from the EDE-Q due to lack of multi-item measures evaluating the construct (e.g., [6, 31]. Given that the four-item measure has not been validated, future research should explore methods of assessing fear of weight gain. The course of CBT-E implemented in our trial was also relatively short (12 sessions, perhaps a longer duration of treatment would have resulted in greater decreases in fear of weight gain.

CBT-E appears to implicitly target fear of weight gain during treatment of binge-spectrum EDs likely through regularizing eating patterns, reducing compensatory behaviors, and breaking dietary rules. We also observed that fear of weight gain predicted more frequent LOC episodes, suggesting that fear of weight gain is a worthy target for future interventions for BN and BED. Clinicians may find it helpful to explicitly discuss fear of weight gain with patients and to make reducing this fear a direct aim of treatment, especially for those experiencing more intense fear. Additional research is needed to understand whether fear of weight gain predicts use of compensatory behaviors in those with BN. Furthermore, future research should investigate whether an intervention explicitly designed to target fear of weight gain (e.g., exposure therapy) would produce even greater changes in fear than CBT-E, and whether reductions in fear result in decreases in LOC episodes and compensatory behaviors. Finally, longitudinal studies must examine whether decreases in fear of weight gain during treatment have an effect on long-term remission from EDs.

### What is already known on this subject?

Research has established that fear of weight gain is a core symptom in eating disorders, including for those with bulimia nervosa and binge eating disorder [5]. Little is known about whether the current gold-standard treatment for eating disorders, CBT-E, targets fear of weight gain. Additionally, research has yet to examine whether fear of weight gain is directly associated with binge episodes.

### What does this study add?

Our study demonstrates that CBT-E does result in decreases in fear of weight gain across treatment. Those with higher fear of weight gain experience more LOC episodes in the following week, suggesting there may be a causal link between fear of weight gain and LOC eating.

## Data Availability

The datasets generated and analyzed during the current study are available from the corresponding author on reasonable request.

## Footnotes

1 1 Fear of weight gain and fear of fatness have been used somewhat interchangeably in the literature to describe weight-related fears. We employed the term *fear of weight gain* throughout this paper to refer to this concept.
